# Effect of different spiking procedures on the distribution and toxicity of ZnO nanoparticles in soil

**DOI:** 10.1007/s10646-012-0914-3

**Published:** 2012-05-03

**Authors:** Pauline L. Waalewijn-Kool, Maria Diez Ortiz, Cornelis A. M. van Gestel

**Affiliations:** 1 Department of Animal Ecology, Faculty of Earth and Life Sciences, VU University, De Boelelaan 1085, 1081 HV Amsterdam, The Netherlands; 2 Centre for Ecology and Hydrology, Maclean Building, Benson Lane, Crowmarsh Gifford, Wallingford, Oxfordshire OX10 8BB UK

**Keywords:** Zinc oxide nanoparticles, Soil, Spiking, *Folsomia candida*, Ecotoxicity

## Abstract

Due to the difficulty in dispersing some engineered nanomaterials in exposure media, realizing homogeneous distributions of nanoparticles (NP) in soil may pose major challenges. The present study investigated the distribution of zinc oxide (ZnO) NP (30 nm) and non-nano ZnO (200 nm) in natural soil using two different spiking procedures, i.e. as dry powder and as suspension in soil extract. Both spiking procedures showed a good recovery (>85 %) of zinc and based on total zinc concentrations no difference was found between the two spiking methods. Both spiking procedures resulted in a fairly homogeneous distribution of the ZnO particles in soil, as evidenced by the low variation in total zinc concentration between replicate samples (<12 % in most cases). Survival of *Folsomia candida* in soil spiked at concentrations up to 6,400 mg Zn kg^−1^ d.w. was not affected for both compounds. Reproduction was reduced in a concentration-dependent manner with EC50 values of 3,159 and 2,914 mg Zn kg^−1^ d.w. for 30 and 200 nm ZnO spiked as dry powder and 3,593 and 5,633 mg Zn kg^−1^ d.w. introduced as suspension. Toxicity of ZnO at 30 and 200 nm did not differ. We conclude that the ZnO particle toxicity is not size related and that the spiking of the soil with ZnO as dry powder or as a suspension in soil extract does not affect its toxicity to *F. candida*.

## Introduction

As nanotechnology industry evolves and “nanotechnology enhanced” products enter the consumer world, environmental exposures are an inevitable consequence. Releases of nanoparticles (NP) may negatively affect the environment and toxic effects on organisms living in soils or water may occur. Ecotoxicological data are needed to establish sound risk assessment for this class of substances. The use of manufactured NP is a relatively new area of science and technology and the first papers on NP and ecotoxicity were published in 2006 (Kahru and Dubourguier 2010).

The nano-size of these particles results in specific physicochemical characteristics that may differ from those of the bulk substance or larger sized particles. The high surface area in relation to the volume of the particle is the main cause of these differences. When particle size decreases the particle surface area increases exponentially. These characteristics may result in favourable properties for use in e.g. new cosmetic products, such as transparent sunscreens instead of the normal white ones. ZnO-NP is used in a wide range of cosmetic products such as foundations, hand creams and as a UV absorber in sunscreens. The physicochemical properties on the other hand also can lead to complex interactions of the (small) particles with the test media used in ecotoxicology. Test organisms therefore may not be exposed to the pristine NP powder or suspension as provided by a commercial supplier. Due to insolubility of NP in water and their aggregation in exposure media test organisms may be exposed to other forms or sizes of NP than were initially added to the test system (Heckmann et al. 2011). The behaviour of NP in soils is a complex process, due to their aggregation/agglomeration and abiotic factors affecting these processes such as pH, CEC and natural organic matter (NOM) content (Quik et al. 2010). In order to better understand how the presentation of NP in toxicity tests affects their uptake and toxicity, it is important to know the way NP interact with components of the test media.

The spiking of test media with NP for standardized ecotoxicity testing is an extremely important first step, because homogeneity of NP distribution in test media is difficult to establish and may influence the outcome of the toxicity test. Currently, NP are introduced in aquatic and terrestrial systems as dry powders or as suspensions, in stabilizing solvents, with or without sonication. Laban et al. (2010) compared stirring and sonication of Ag-NP solutions and found no difference in the percentage of dissolved Ag released in an aquatic test system. Some studies report the introduction of metal NP in soil as dry powder (Hu et al. 2010; Manzo et al. 2011) while others applied NP to the soil as a suspension in deionised water (Heckmann et al. 2011; Shoults-Wilson et al. 2011). Van der Ploeg et al. (2011) introduced C60 NP into soil, dissolved in an aqueous soil extract. The extract was obtained by stirring control soil in water. After filtering, C60 was added to the extract and the suspensions obtained were stirred to acquire an as stable and homogeneous NP suspension as possible. This spiking method can influence the soil–water partitioning of the NP and in case of metal-based NP, substantial metal ions can be released from the NP. Further investigation of different spiking methods of NP is needed in order to develop a uniform approach for testing NP. It also remains uncertain to what extent the outcome of a toxicity test is influenced by the spiking method.

This study aimed at comparing different spiking methods, using ZnO-NP. Characterization of NP in soil in general is rather difficult. Instead, total soil analyses and toxicity tests may provide insight into NP distribution in soil and potentially toxic effects that are related to NP concentration. ICP-MS and ICP-AES showed good recoveries (80–120 %) for aluminium using soil spiked with dry Al_2_O_3_ NP powder (Coleman et al. 2010). And microwave digestion of soil spiked with Ag-NP (suspension in deionised water) showed 100 % recovery on average (Shoults-Wilson et al. 2011).

The springtail *Folsomia candida* (Collembola) has widely been used to assess the environmental impact of a range of pollutants on soils due to their abundance and diversity. *F. candida* is a parthenogenetic species and the rate of reproduction makes this species suitable for studying the effects on reproduction (Fountain and Hopkin 2005). A widely-stated hypothesis is that nano-sized particles are more potent than larger particles of the same nominal substance because of their increased surface area per unit mass. This has been supported by animal studies with carbon black (Jia et al. 2005). Studies with metal-based NP found no clear relationship between NP size as such and the effect on soil organisms (Unrine et al. 2010).

The present study investigates the spiking of natural soil with differently sized ZnO particles (30 and 200 nm) using two different procedures, i.e. as dry powder and as a suspension in soil extract. Five sub-samples of spiked soil were analyzed to evaluate the distribution of zinc in the treated soil, and porewater samples were taken to evaluate possible differences in Zn dissolution. Differences in toxicity between the spiking methods and the particles were evaluated by exposing the springtail *F. candida* to the spiked soil. After four weeks exposure the effects on the survival and reproduction were determined and toxicity values (EC50/EC10) were estimated based on total zinc concentration in the soil.

## Materials and methods

### Nanoparticle characterization and spiking procedures

**Fig. 1:**
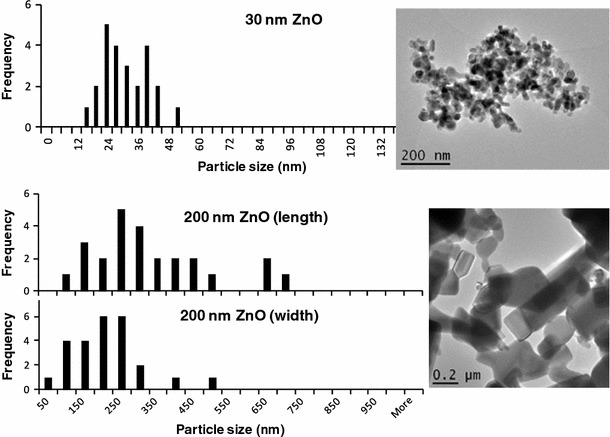
Primary Particle Size Distribution (*left*) from TEM (*right*) of 30 nm ZnO (*top*) with primary particle sizes of approx. 30–50 nm, and of 200 nm ZnO (*bottom*) with primary particle sizes of approx. 50–500 nm. Primary particles were dispersed in deionized water, sonicated and deposited on a carbon coated Cu TEM grid

Two sizes of zinc oxide (ZnO) were applied, namely 30 nm (Nanosun ZnO P99/30) and 200 nm (Microsun ZnO W45/30). Figure 1 shows transmission electron micrographs (TEM) of the ZnO powders, which were dispersed in deionized water. Solutions were sonicated in a low power ultra sonication bath for 30 s and 1 drop (20 μl) was deposited on a carbon coated Cu TEM grid. Samples were dried at room temperature for several hours before examination in the TEM. Experiments were carried out on a JEOL 2010 analytical TEM, which has a LaB_6_ electron gun and can be operated between 80 and 200 kV. This instrument has a resolution of 0.19 nm, an electron probe size down to 0.5 nm and a maximum specimen tilt of ±10 ° along both axes. The instrument is equipped with an Oxford Instruments LZ5 windowless energy dispersive X-ray spectrometer (EDS) controlled by INCA. On the TEM images a small (approx. 20–25) number of particles were measured from about 4 or 5 TEM micrographs to get a rough particle size distribution for the primary particles. Each particle was measured individually from the TEM micrographs using Digital Micrograph program, which is a standard TEM instrument control and analysis program. The TEM images show that the ZnO particles were mainly equiaxial and rounded. Figure 1 shows the primary particle size distribution of the ZnO particles from the TEM images.

Seven concentrations (nominal range 100–6,400 mg Zn kg^−1^ dry soil) and two controls without ZnO were tested. Loamy sand soil (LUFA-Speyer 2.2, Sp 2121, Germany, 2009) with a reported ${\text{pH}}_{{{\text{CaCl}}_{ 2} }}$ of 5.5, a total organic carbon content of 2.09 %, a cation exchange capacity (CEC) of 10.0 cmol_c_ kg^−1^ and a water-holding capacity (WHC) of 46.5 % was used. For the dry spiking, powders were mixed in with 20 g dry soil in glass jars. The treated soil was added to 180 g dry soil in glass containers. After mixing, water was added to reach 50 % of the WHC. Soil–water suspensions were prepared by mixing air-dried soil with Milli-Q using a soil–water ratio of 2:5 (w/v) (van der Ploeg et al. 2011). The suspensions were shaken at 180 rpm at ambient temperature for one hour and filtered over a filter paper under vacuum. ZnO powders, corresponding with the nominal concentrations, were weighed and added to 30 ml of the filtrates. Soil solutions were shaken for two days at 180 rpm. Then, suspensions were mixed in with 200 g dry soil to obtain nominal test concentrations. Also with this procedure soil was moistened to 50 % of its WHC. The use of soil extract as spiking solution may prevent the ZnO-NP to form aggregates and may diminish settlement or flocculation.

To visualize the particle size and the degree of aggregation of ZnO particles in the spiking solution, images of the spiking solution (approx. 500 mg l^−1^) were taken using transmission electron microscope (TEM) operating at 60 kV (JEOL 101, containing a 2 k CCD camera). Samples were dropped on a 75 mesh copper Formvar grid and left to dry before examination by TEM. The particle size distribution in the spiking solutions were analysed by nanoparticle tracking analysis (NTA) using NanoSight LM20 (size range 10–1,000 nm). Videos were analyzed using NTA software (Version 1.5) and the minimum required tracks of 100 were completed per video analysis. Therefore, spiking solutions were 100 times diluted in Milli-Q water and followed in real-time for 90 s.

### Zinc analysis in the soil and pore water

Five samples per treatment (±100 mg dried soil) were randomly taken from the batches of spiked soil and digested in a mixture of Milli-Q, concentrated HCl and concentrated HNO_3_ (1:1:4 by vol.) using an oven (CEM MDS 81-D). After digestion for 7 h at 140 °C, solutions were analysed for total zinc concentration by flame atomic absorption spectrometry (AAS) (Perkin-Elmer 1100B). Certified reference material (ISE sample 989 of River Clay from Wageningen, The Netherlands) was used to ensure the accuracy of the analytical procedure. Measured zinc concentrations in the reference material were within 10 % of the certified concentrations. A two-sided Student *t* test was performed to compare spiking methods for each concentration.

Soil pore water was collected by centrifuging 50 g soil (Centrifuge Falcon 6/300 series, CFC Free), after saturation with Milli-Q and one week equilibration. Soils were centrifuged for 50 min with a relative force of 2,000 g over a round filter and a membrane filter (Whatman 0.45 μm), placed inside the tubes (method described by Hobbelen et al. 2004). Approximately 10 ml pore water per sample was collected for analysis by flame AAS (Perkin-Elmer 1100B). Zinc concentrations in the samples were also determined by flame AAS after ultrafiltration of the soil pore water to obtain a particle-free extract. For this, soil solutions were centrifuged in a 100 kDa ultrafiltration device (Amicon Ultra-15 Filters, Millipore) for 20 min at 2,000 g.

### Toxicity test with *F. candida*

The springtail *F. candida* (“Berlin strain”; VU University Amsterdam) was cultured in pots with a base of moist plaster of Paris mixed with charcoal at 20 ± 1 °C at a light/dark regime of 12/12 h. The experiment was initiated with juveniles of the same age (10–12 days) that were obtained by synchronising the egg laying of the culture animals, fed with dried baker’s yeast (Dr. Oetker). Toxicity tests were performed following ISO guideline 11267 (ISO 1999). The test was conducted in 100 ml glass jars containing 30 g moist soil. Five replicates for each zinc concentration and control were prepared. At the start of the test, ten synchronised animals were transferred into each test jar. The test jars were filled randomly and before introduction the animals were checked under the microscope for a healthy appearance. The animals were fed a few grains of dried baker’s yeast. The jars were incubated in a climate room at 20 ± 1 °C and with a 12/12 h light/dark cycle. Once a week, the moisture content of test soils was checked by weighing the jars, and moisture was replenished with Milli-Q when necessary. The jars were also aerated by this procedure.

After four weeks, the jars were sacrificed for determination of springtail survival and reproduction. Each jar was emptied into a 200 ml beaker glass and 100 ml Milli-Q was added. The mixture was stirred carefully to let all the animals float to the surface. After the adults were counted by eye, a picture of the water surface was taken using a digital camera (Olympus, C-5060). The Cell^^D^ imaging software was applied to count the number of juveniles for determining the effect on reproduction. EC50 values, the actual concentration in the soil causing a 50 % reduction in reproduction, were estimated applying a logistic model according to Haanstra et al. (1985). In addition, 10 % effective concentration (EC10) values were obtained by modifying the logistic model. A generalized likelihood ratio test (Sokal and Rohlf 1995) was applied to compare EC50 values obtained for both ZnO particles and for both spiking procedures. All calculations were performed in SPSS Statistics 17.

## Results

### Zinc distribution in the spiking solution

**Fig. 2:**
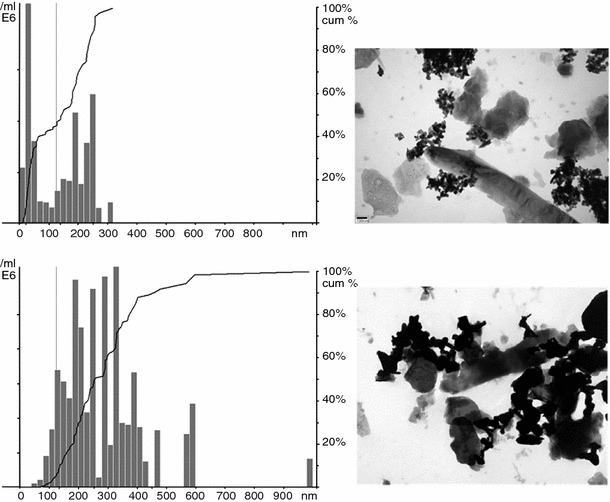
Particle Size Distributions determined by NTA (*left*) and TEM (*right*) of the Lufa 2.2 soil extract spiked with 30 nm (*top*) and 200 nm ZnO (*bottom*). For the particle size distribution analysis, extracts spiked at 500 mg Zn l^−1^ were 100 times diluted in Milli-Q to obtain concentrations of approx. 5 mg Zn l^−1^. The line represents cumulative particle sizes as a percentage of the total. The TEM images are from the non-diluted soil extracts (500 mg Zn l^−1^), and show aggregates of ZnO particles and the binding of aggregates to dissolved NOM

Figure 2 shows TEM of Lufa 2.2 soil extract with 30 and 200 nm ZnO, used for the spiking solution to reach 100 mg Zn kg^−1^ dry soil. Figure 2 clearly shows that the ZnO particles form aggregates in the soil extract. Free ZnO particles, as shown in the TEM images (Fig. 1), were not observed in the samples. All aggregated ZnO particles were bound to NOM in the soil extract. Aggregated ZnO particles seemed not to occupy all the empty spaces available along the NOM (Fig. 2). Attractive forces and binding of particles to each other can not be avoided by the addition of NOM to the spiking solution. NTA of the ZnO particles in the spiking solutions confirmed that ZnO particles were aggregated in the spiking solutions, but the histograms indicate a clear difference between the two ZnO particles (Fig. 2). The NTA shows that the 30 nm ZnO has a strong peak for the primary particle size and aggregates/agglomerates in the 100–300 nm size range. The 200 nm ZnO, however, does not have a strong peak below approx. 100 nm, and the particle size range is much broader (200–600 nm).

### Zinc concentrations in soil and pore water

Both spiking procedures, i.e. dry powder and suspension spiking, showed a recovery above 85 % of zinc in Lufa 2.2 soil (Table 1). Zinc concentrations in the soil were corrected for the zinc measured in the controls. The analyses of five subsamples, randomly taken from the treated soil, showed that spiking with dry powder or suspension did not influence the distribution of ZnO particles in the soil. Based on these values no difference between the spiking procedures was found. Both spiking procedures resulted in a fairly homogeneous distribution of the ZnO particles in soil, as evidenced by the low variation between replicate samples (<12 % in most cases). The results of the two-sided Student *t* test showed significant differences in total Zn concentrations in the soil between spiking methods in some cases, but differences were not consistent and not concentration related.

**Table 1:** Average zinc concentrations (±SD, *n* = 5) measured in Lufa 2.2 soil spiked with 30 and 200 nm ZnO as dry powder (D) and as suspension in soil extract (S)

Nominal concentrations (mg Zn kg^−1^ d.w.)	30 nm, D	30 nm, S	200 nm, D	200 nm, S
Control	21.2 ± 2.1	17.9 ± 0.1	21.2 ± 2.1	17.9 ± 0.1
100	133 ± 15.5 (133)	122 ± 5.7 (122)	150 ± 15.3 (150)*	121 ± 11.0 (121)*
200	223 ± 7.97 (112)*	195 ± 13.3 (97.3)*	258 ± 15.7 (129)*	218 ± 13.5 (109)*
400	438 ± 17.4 (109)*	475 ± 11.0 (119)*	435 ± 18.9 (109)	458 ± 38.1 (115)
800	864 ± 41.2 (108)	904 ± 85.4 (113)	908 ± 65.4 (114)	993 ± 102 (124)
1,600	1,598 ± 112 (99.9)*	1,899 ± 223 (119)*	1,813 ± 318 (113)	1,575 ± 62.2 (98.5)
3,200	3,280 ± 175 (103)	3,322 ± 369 (104)	2,913 ± 478 (91.0)	3,075 ± 344 (96.1)
6,400	5,502 ± 274 (86.0)	5,684 ± 967 (88.8)	6,787 ± 205 (106)*	5,608 ± 390 (87.6)*

Zinc concentrations in the soil are corrected for the zinc levels measured in the controls. Recoveries (%) are presented in brackets. A significant difference (* *P* < 0.05) between the spiking methods for each concentration, using a two-sided Student *t* test

Zinc concentrations in the soil pore water were corrected for the zinc levels in the controls and ranged from 3.32 to 8.22 mg Zn l^−1^ for the lowest spiking concentration and from 18.3 to 25.8 mg Zn l^−1^ for the highest one (Table 2). No linear trend was observed between zinc concentrations in the soil pore water and the actual zinc concentrations in Lufa 2.2 soil. The maximum soluble zinc concentration was reached at intermediate spiking concentrations for both spiking procedures. Zinc concentrations in the pore water of soil spiked with ZnO as dry powder were slightly higher than the ones for suspension-spiked soils. This may suggest that ZnO-NP (or dissolved zinc) from suspensions are less available in the soil pore water due to binding of zinc to organic matter in the suspensions. However, the difference in soluble zinc is negligible based on measured total zinc concentrations in the soil (max. 1.24 % for dry spiking and max. 0.841 % for suspension spiking). Ultrafiltration did not reduce zinc concentrations in the porewater samples (Table 2), suggesting all zinc was available. The higher zinc concentrations after ultrafiltration in some samples are considered an experimental artefact.

**Table 2:** Zinc concentrations measured (*n* = 1) in the pore water of Lufa 2.2 soil spiked with 30 and 200 nm ZnO as dry powder (D) and as suspension in soil extract (S) expressed as mg Zn l^−1^

Nominal concentrations (mg Zn kg^−1^ d.w.)	30 nm, D	200 nm, D	30 nm, S	200 nm, S
Control	0.60 (0.35)	0.60 (0.35)	0.63 (0.47)	0.63 (0.47)
100	6.78 (6.80)	8.22 (7.54)	4.55 (4.37)	3.32 (3.01)
200	12.0 (11.6)	11.6 (12.0)	6.98 (7.60)	5.99 (6.01)
400	20.9 (20.2)	23.8 (25.1)	15.9 (14.7)	14.1 (11.0)
800	21.8 (27.8)	21.8 (28.3)	19.5 (16.4)	15.7 (12.4)
1,600	22.1 (26.9)	24.5 (31.7)	20.2 (25.6)	20.4 (19.3)
3,200	23.8 (24.4)	25.8 (30.1)	18.3 (22.6)	18.6 (19.3)
6,400	21.7 (24.9)	24.7 (24.6)	18.4 (21.6)	22.2 (24.1)

Soil pore water was collected one week after saturation of the soils with Milli-Q. Zinc concentration in the control was deducted from the zinc concentrations measured in the soil pore water of the different treatments. Zinc concentrations measured in the soil pore water after ultrafiltration are presented in brackets

### Toxicity to *F. candida*

**Fig. 3:**
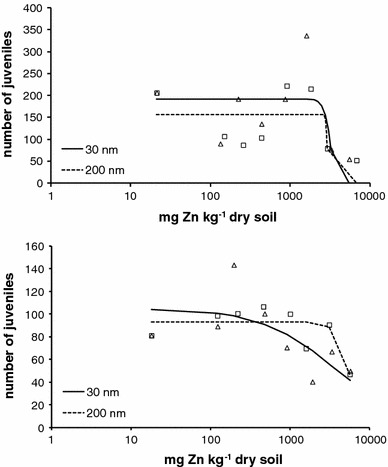
Effect of 30 nm (*open triangle*) and 200 nm (*open square*) ZnO on the reproduction (average number of juveniles per test concentration is shown) of *F. candida* after 28-day exposure in Lufa 2.2 soil. Actual exposure concentrations of zinc in the soil are provided on the *x*-axis applying suspension spiking (*top*) and dry spiking (*bottom*). Lines show fit obtained with a three parameter logistic dose–response model: ymax/(1 + (concentration/EC50)^b^). The resulting equations are for 30 nm suspension spiking: 105/(1 + (conc./3,593)^0.973^ (*r* ^*2*^ = 0.140), for 200 nm suspension spiking: 93/(1 + (conc./5,633)^4.94^ (*r* ^*2*^ = 0.122), for 30 nm dry spiking: 192/(1 + (conc./3,159)^10.4^ (*r* ^*2*^ = 0.258) and for 200 nm dry spiking: 157/(1 + (conc./2,914)^43.2^ (*r* ^*2*^ = n.a.)

Survival of *F. candida* in soil spiked at concentrations up to 6,400 mg Zn kg^−1^ was not affected for both ZnO powders. The average numbers of juveniles in the two control soils were 206 (±91.7, *n* = 5) for dry spiking series and 81 (±45.1, *n* = 5) for suspension spiking. The effect of 30 and 200 nm ZnO particles on reproduction was concentration-dependent (Fig. 3) with a steeper dose–response curve for dry spiking than for suspension spiking. The EC50 values for the effect on the reproduction of *F. candida* of 30 and 200 nm ZnO were 3,159 (95 % confidence interval: 126–5,502) and 2,914 (1,813–6,787) mg Zn kg^−1^ for the dry powder series. For the suspension-spiked soils, the EC50s were 3,593 (122–5,684) and 5,633 (3,711–5,608) mg Zn kg^−1^ for 30 and 200 nm ZnO. Large 95 % confidence intervals were estimated and no significant differences between the EC50 values were found when applying a generalized likelihood-ratio test (χ^2^ < 3.84; n.s.). Corresponding EC10 values were 2,559 (133–5,502) mg Zn kg^−1^ for the 30 nm ZnO particles and 2,730 (149–6,787) for the 200 nm particles spiked in the soil as dry powder. For the 200 nm particles spiked as suspension EC10 was 3,611 (121–5,608) mg Zn kg^−1^. No EC10 could be calculated for the 30 nm ZnO particles spiked as suspension. EC50 values based on porewater concentrations could not be estimated due to the small differences in zinc concentrations measured in the pore water.

## Discussion

Our study aimed at comparing two spiking procedures in order to find the most suitable method to introduce nanoparticle powders in soil ecotoxicological tests. Regarding the soil sample preparation for toxicity testing it may not be suitable to prepare the dosing using a dilution series of NP. Starting at the highest concentrations and diluting until the lowest test concentration may cause high deviations in the samples due to agglomeration of NP at each dilution step. The dosing is performed more precise when the amount of particles needed (weight in case of powders or volumes in case of suspensions) is added to the soil or other type of media for each test concentration separately.

Assessing NP or particle distribution in soil is difficult. In this study, we therefore focused on assessing the NP distribution in the soil extract used for suspension spiking. We showed that NTA analysis can be used for assessing particle size distributions in soil extracts. This technique, and many other characterization methods, provides the analysis at one time point and for one test concentration. In this study only one concentration (i.e. 5 mg Zn l^−1^) has been analyzed at one time point. This concentration was 100 times lower than the Zn concentration in the spiking solution applied to reach the lowest concentration tested of 100 mg Zn kg^−1^ soil. Figure 2 shows one possible situation of the behaviour of ZnO particles in soil solution. Further in-depth characterization studies are necessary to assess NP behaviour (aggregation/agglomeration, particle size distributions) in soil and/or soil solution.

Instead of characterizing particle distribution in the soil, we analysed the soil for total Zn concentrations. The analyses of five subsamples, randomly taken from the treated soil, showed that spiking with dry powder or suspension did not influence the distribution of ZnO particles in the soil. The study showed good recoveries (>85 %) at all test concentrations with the ZnO particles. Both spiking procedures are currently applied in ecotoxicity testing and the most appropriate method has not been established yet among ecotoxicologists. A disadvantage of dry spiking is the static force of the particles that make them easily blown away. On the other hand dry particles are easily mixed in with dry soil particles. Introduction of NP in a soil extract may prevent agglomeration or flocculation as particles bind to NOM or minerals. This procedure however, works better for dissolved NP than for insoluble particles.

The results obtained in this study showed that the survival of *F. candida* was not affected by ZnO particles up to 6,400 mg Zn kg^−1^ d.w. The effect on reproduction was found to be concentration related and the EC50 values of the two compounds were not significantly different. Theoretically, it is expected that toxicity would be higher for 30 nm ZnO compared to 200 nm ZnO due to the larger surface area per volume, the NP therefore having more reactive sites to induce potential biological effects. Also, due to their small size ZnO-NP might have both greater mobility as well as potentially enhanced uptake across biological membranes. However, it seems that the size of ZnO does not contribute to a significant difference in the effect observed on springtail reproduction. This is in line with an aquatic toxicity study in which a 72-h IC50 of 60 mg Zn l^−1^ was found for the effect of both ZnO-NP (30 nm) and bulk ZnO on the growth of the freshwater micro-algae *Pseudokirchneriella subcapitata* (Franklin et al. 2007). Wiench et al. (2009), testing different nano and non-nano ZnO particles, reported that the toxicity for *Daphnia magna* was independent of particle size, although the EC50 values for ZnO-NP and non-nano ZnO differed by a factor of 8. Also, Kool et al. (2011) showed that ZnO-NP and non-nano ZnO were equally toxic to *F. candida* in soil with reported EC50s of 1,964 and 1,591 mg Zn kg^−1^ d.w. The characterization of particles in the spiking solution shows that the original ZnO particles were not present in their pristine form after their introduction into the soil extract (Figs. 1, 2). A wide range of particles sizes were present in the soil solution and therefore the relation between particle sizes and toxic effect could not be established.

The toxicity of 30 and 200 nm ZnO, based on total zinc concentrations in the soil, was lower than in our previous study with *F. candida*. ZnO-NP are commercially available as dry powders with particle sizes ranging from approximately 20 to 200 nm (Wang 2004). The ZnO particles tested in this study were obtained from a different source (Nanosun ZnO-NP; Microsun non-nano ZnO) compared to the ones used in our previous study (BASF Z-COTE^®^ ZnO-NP and non-nano ZnO from Merck). NP manufactured in different laboratories may have slightly different sizes and physicochemical properties, such as surface charge, size and shape. It is therefore likely that the use of different ZnO-NP has resulted in slightly different toxicities.

Our previous study, in which ZnCl_2_ was tested as a proxy for zinc ions, showed that toxicity is caused by dissolved zinc rather than ZnO-NP as such, because the EC50 values based on soil porewater concentration were in the same range (7.94–16.8 mg Zn l^−1^) (Kool et al. 2011). In this study porewater concentrations did not increase with increasing spiking concentrations, but showed a maximum of 20–25 mg Zn l^−1^. Contrary to our previous study, toxicity therefore cannot fully be explained by dissolved zinc measured in the pore water. The relationship between zinc concentrations in the soil and in the pore water suggests that a particle effect of ZnO can not be excluded. Here, we show that ZnO-NP as such may have an effect on *F. candida* at concentrations between 1,600 and 6,400 mg Zn kg^−1^. The apparent contradiction with our previous study could have been caused by the use of different ZnO particles. More studies are needed to gain insight into the processes occurring in the soil and pore water, in order to establish the Zn exposure to the animals.

This study shows that spiking with dry powder or suspension does not influence the distribution of ZnO-NP in soil and their toxicity to *F. candida*. Both 30 and 200 nm ZnO particles were equally toxic to *F. candida* after 28 days exposure in natural soil.
